# Preparation and Evaluation of Novel *In Situ* Gels Containing Acyclovir for the Treatment of Oral Herpes Simplex Virus Infections

**DOI:** 10.1155/2014/280928

**Published:** 2014-03-24

**Authors:** Binu Chaudhary, Surajpal Verma

**Affiliations:** School of Pharmaceutical Sciences, Lovely Professional University, Phagwara Punjab 144411, India

## Abstract

The objective of this work was to develop an oral mucosal drug delivery system to facilitate the local and systemic delivery of acyclovir for the treatment of oral herpes infection caused by the herpes simplex virus (HSV). An *in situ* gelling system was used to increase the residence time and thus the bioavailability of acyclovir in oral mucosa. Temperature and pH trigged *in situ* gel formulations were prepared by cold method using polymers like poloxamer 407, carbopol 934, and HPMC. Glycerin and a mixture of tween 80 and ethanol (1 : 2 ratio) were used as the drug dissolving solvent. The pH of carbopol containing formulation was adjusted to pH 5.8 while the pH of poloxamer solution was adjusted to pH 7. These formulations were evaluated for sol-gel transition temperature, gelling capacity, pH, viscosity, spreadability, gel strength, drug content, ex-vitro permeation, and mucoadhesion. The gelation temperatures of all the formulations were within the range of 28–38°C. All the formulations exhibited fairly uniform drug content (98.15–99.75%). Drug release study of all the formulations showed sustained release properties. The release of drug through these *in situ* gel formulations followed the Higuchi model and Korsmeyer peppas model mechanism.

## 1. Introduction

Acyclovir (ACY), a widely used antiviral agent, is a synthetic purine nucleotide analog derived from guanine. It is effective in the treatment of herpes simplex virus (HSV), mainly HSV 1 and HSV 2, and varicella zoster [1, 2]. Acyclovir exerts its antiviral activity by competitive inhibition of viral DNA through selective binding of acyclovir to HSV-thymidine kinase [3, 4]. Currently available dosage forms of acyclovir are intended for intravenous, oral, and topical administration. Systemic delivery of the drug following administration by these routes is far from optimal. However, the oral absorption of acyclovir is dose dependent and highly variable with bioavailability ranging from 15 to 30% [5, 6].

The percutaneous penetration is poor; and because of its limited solubility in water (1.62 mg/mL at 22°C) it cannot be given as eye drops or by intramuscular injection. As parenteral administration acyclovir is presently available as infusion or as bolus intravenous injection in the form of strong alkaline (pH 10-11) solution of sodium salt. Consequently, administration of this dosage form may cause thrombophlebitis or perivascular inflammation [5].

There have been many attempts for improving the physicochemical properties of acyclovir by chemical modifications which are documented in the literature. These include using acyclovir as prodrug, employing novel redox-based chemical targeting systems to enhance ocular, parenteral, nasal, and intradermal delivery of drug for greater oral bioavailability. Because of its poor oral and transdermal absorption of acyclovir, oral mucosa is a logical choice for local and systemic delivery [6, 7]. There are several reasons due to which oral mucosa is an attractive site for the delivery of therapeutic agents that include its accessibility, excellent blood supply, by-pass of hepatic first-pass metabolism, rapid repair, and permeability profile [8].

The limitation of oral mucosal drug delivery is the dilution and rapid elimination of topically applied drugs due to the flushing action of saliva. The delivery system in which the drug is incorporated is therefore an important consideration and should be formulated to prolong the retention of the drug in the oral cavity. Bioadhesive polymers have been utilized in gel forms to prolong the residence time on oral mucosa and to reduce the frequency of application and the amount of drug administered. This might improve patient's compliance and acceptance of the drug product [9].

*In situ* is a Latin phrase which can be translated literally as “In process.”* In situ* gels are drug delivery systems that are in solution forms before administration in the body, once administered they undergo gelation* in situ* to form a gel. It is basically a polymeric drug delivery system.

Administration routes for* in situ* gels are oral, ocular, rectal, vaginal, injectable, and intraperitoneal. Advantages of* in situ* forming mucoadhesive polymeric delivery systems include ease of administration, improved local bioavailability, reduced dose concentration, reduced dosing frequency, and improved patient compliance and comfort. Also the formulation is less complex which lowers the investment and manufacturing cost [10]. There are several possible mechanisms that lead to* in situ* gel formation: solvent exchange, UV irradiation, ionic cross-linkage, pH change, and temperature modulation [11].

The aim of this study was to develop an* in situ* gel formulation containing acyclovir for local and systemic delivery from oral mucosal route. This was needed to increase absorption of the drug leading to an improvement in its bioavailability, to reduce its dosing frequency, and to achieve sustained release effect.

## 2. Materials and Methods

### 2.1. Materials

Acyclovir was purchased from Jackson Laboratories (P) Ltd., India. Poloxamer 188 and poloxamer 407 were received as gift samples from BASF chemical company, India. Carbopol 934, methyl paraben, and propyl paraben were purchased from Central Drug House (P). Ltd. India. HPMC K-100, glycerin, and ethanol were procured from LOBA Chemie pvt, Ltd., India. Tween 80 and triethanolamine were obtained from Molychaem, India.

### 2.2. Solubility Studies

The solubility of acyclovir was studied in water and in buffer solutions of different pH. These were HCl buffer (pH 1.2), acetate buffer (pH 4.5), phosphate buffer (pH 6.8 and 5.5), and borate buffer (pH 9.8). For evaluating the solubility in a particular solvent, an excessive amount of the drug was dissolved in 5 mL solvent and the solution was stirred using magnetic stirrer for 24 hrs at room temperature (25°C). After 24 hrs the sample was removed from stirrer and allowed to settle down. The supernatant solution was separated and filtered, and appropriate dilution was made with the respective solvents. Absorbance of diluted solution was measured at 255 nm and the concentration of soluble drug was calculated.

Similarly, the solubility of acyclovir was studied in surfactants like tween 80, tween 20, and SLS and cosurfactants like propylene glycol, PEG 400, and glycerin. Solubility was also studied in oleic acid, castor oil, and the mixture of tween 80 and ethanol (1 : 2 ratio). In this case solubility study was conducted for 48 hrs at 37 ± 5°C.

### 2.3. Preparation of* In Situ* Gel Formulation

*In situ* gel was prepared by the cold method. A weighed amount of poloxamer 407 and poloxamer 188 (15–20% w/v) was slowly added to 15 mL water (at 4 ± 2°C) in a beaker with continuous stirring using a magnetic stirrer at a speed of 500 rpm for 2 hrs. The temperature of water was maintained at 4 ± 2°C throughout the preparation. This solution was kept overnight in refrigerator. HPMC K-100 (0.5% w/v), carbopol 934 (0.1%, 0.3%, and 0.5% w/v), and the preservatives (methyl and propyl paraben 0.1% and 0.01%, w/v resp.) were added to poloxamer dispersion with continuous stirring. The preservative solution was prepared by solubilizing it in hot water. It was mixed with above dispersion after cooling. The weighed amount of drug (2% w/v) was dissolved in the mixture of tween 80 and ethanol (1 : 2) or glycerin. The drug solution was then mixed in the above described poloxamer dispersion. The final volume was made up and pH of the poloxamer dispersion was adjusted to 7 using triethanolamine, whereas the dispersion containing carbopol was adjusted to pH 5.8. The composition of the* in situ* gel formulations is shown in Tables 1 and 2 [12, 13].

### 2.4. Determination of Sol-Gel Temperature (*T*_**sol-gel**_)

The gelation temperature was determined by placing the solution in test tube: the test tube was dipped in water bath whose temperature was maintained at 37 ± 5°C for 2 min. The temperature at which solution was converted to gel was noted down by placing the thermometer in the test tube. In case of formulations containing carbopol, the formulation was taken in test tube containing phosphate buffer solution pH 6.8. This mixture was thoroughly mixed and dipped into the water bath. The maximum limit for gelation was checked up to 60°C. The gel was said to have formed when there was no flow of the formulation when the container was overturned.

### 2.5. Determination of Gelling Capacity

The gelling capacity was determined based on the formulation behaviors like gelling time and erosion time of formed gel due to the environmental changes.

### 2.6. Determination of pH

The pH of the gel was determined using calibrated pH meter. Determinations were carried out in triplicate and an average of these determinations was taken as the pH of the gel.

### 2.7. Viscosity of Formulation at Solution State and Gel State

The viscosity was measured at 25°C and 37°C using Brookfield viscometer and spindle number 62 at 100 rpm. First, the viscosity of gel solution was measured. This solution was allowed to convert to gel by increasing the temperature of the solution with the help of water bath whose temperature was maintained at 37 ± 1°C. In the formulation containing carbopol pH was increased along with temperature. Then the viscosity of this formed gel was measured. The average of two determinations was taken.

### 2.8. Spreadability Test

To determine the spreadability of the gel, approximately 1 g of gel was placed at the center of the glass plate (20 cm × 20 cm). This glass plate was covered with another glass plate of the same size. Next, the weight of 1000 g was carefully applied on the upper side of the plate; as a result the gel was spread out in between the plates. After one minute the weight was removed and the diameter of the spread area (cm) was measured. This determination was carried out in triplicate [14, 15].

### 2.9. Gel Strength

An accurate weighed quantity of 30 g of gel was placed in a 50 mL graduated measuring cylinder and was allowed to form gel in a water bath at 37°C. By applying 50 g weight to the gel with the help of a cylinder, the time taken by the cylinder to sink 5 cm down through the gel was measured [16].

### 2.10. Drug Content

*In situ* gel formulation, equivalent to 10 mg of acyclovir (0.5 mL), was pipetted out. It was suitably diluted with 0.1 N NaOH solution and the absorbance of this mixture was measured at 255 nm. The drug content was calculated against the absorbance of control ACY solution of the same concentration at 255 nm.

### 2.11. *Ex Vivo* Permeation Study

*Ex vivo* permeation study was assessed by using Franz diffusion cell. The porcine oral mucosa was used as biological membrane for the study. Porcine oral mucosa was obtained from local slaughter house and stored in phosphate buffer (pH 7) at 4°C from the time of collection. It was used within 3 hrs of procurement. The receptor compartment was filled with phosphate buffer (pH 7.4). The compartment also containing magnetic bead for stirring purpose. The suitable size of membrane was placed in between the donor and the receptor compartment. The cell was agitated by magnetic stirrer at 600 rpm and maintained at 37 ± 1°C. Approximately 500 mg of gel as a sample was transferred to the donor compartment. About 3 mL sample was withdrawn at the different time intervals of 15, 30, 45, 60, 75, 90, 120, 180, and 360 min. After each withdrawal, equal volume of fresh phosphate buffer of pH 7.4 previously heated to 37 ± 1°C was incorporated in the receptor compartment to maintain the sink conditions. The samples were filtered and diluted, and their absorbance was measured at 255 nm [17, 18].

### 2.12. Analysis of Release Mechanism

The release kinetics of acyclovir from* in situ* gel formulation was evaluated considering five different models including zero order, first order Higuchi model, Hixson Crowell, and Korsmeyer's peppas model [19].

### 2.13. Mucoadhesion Studies

Mucoadhesive property was determined using modified physical balance. Porcine oral mucosa was used as biological membrane, which was fixed under one pan of the balance with the help of cyanoacrylate glue and was hydrated with 100**μ**L of phosphate buffer pH 6.8 maintained at 37 ± 1°C. Accurately weighed amount of 1 g of gel was stuck to the inverted beaker (250 mL) using glue and the height of the balance was adjusted to accommodate a glass container below the pan where membrane was glued. A preload of 20 g was applied in order to allow the formation of mucoadhesive joints. After a 3 min rest period, the preload was removed and gradually the weight was added to the other pan until the gel was detached from the mucosal surface. The total weight required for the complete detachment of the gel was recorded [13, 20, 21].

## 3. Results and Discussions

### 3.1. Solubility Studies

Solubility of drug in water was found to be 2.35 mg/mL at room temperature (RT). The pH dependent solubility profile in Table 3 indicates that acyclovir had pH dependent solubility. It had higher solubility in solution of basic pH as compared to the solution at acidic pH. It had less solubility towards neutral pH.

The solubility studies were also performed in different surfactants/cosurfactants/oils/mixtures of surfactants and cosurfactants. Tween 80, glycerin, and mixture of tween 80 and ethanol (1 : 2) had higher solubility of ACY than other vehicles. So, glycerin and mixture of tween 80 and ethanol (1 : 2) were used as solvents in formulations. Although ACY had higher solubility in tween 80 in comparison to its mixture with ethanol, its higher viscosity restricted not using this compound for the formulation development.

### 3.2. Evaluation of Gel

A 20% w/v concentration was found to be the optimum concentration for the poloxamer solution to form an* in situ* gel. However, the gelling temperatures were found to be 30°C and above. But this gel was found to have weak mechanical strength and the erosion occurred rapidly. Hence, hydrophilic polymers such as HPMC, carbopol 934, and poloxamer 188 were incorporated in the formulation to overcome the drawback.

### 3.3. Solution to Gel Transition Temperature (*T*_**sol-gel**_)

The gelation temperature of all the formulations was in the range of 28°C to 38°C, while the transition temperature of the formulations containing tween 80-ethanol (Ft) and glycerin (Fg) showed slight difference. The transition temperature of Ft was slightly higher than Fg formulation because ethanol might have increased the transition temperature.

### 3.4. Gelling Capacity

The gelling capacity data of prepared formulations presented in Table 4 represent that the formulations Ft1 and Fg1 had immediate gelation and underwent rapid dissolution. On the other hand the formulations Ft6 and Fg6 had immediate gelation but exist for an hour. This implied that by increasing the concentration of polymer, transition time was decreased and the erosion time of formed gel was increased.

### 3.5. pH of the Formulation

The pH of the formulations was neutral. This indicated the nonirritancy of the formulation in oral cavity. The formulation containing carbopol 934 was slightly acidic and was maintained within pH 5.8 while the pH of poloxamer solution was adjusted to pH 7.

### 3.6. Viscosity of Formulation at Solution State and Gel State

The viscosity was proportional to the concentration of the mucoadhesive polymer in the formulation. All the formulations exhibited quite low viscosity at low temperature. However, upon increasing the temperature, a gel was formed in well-defined temperature and viscosity of the formulation was increased.

### 3.7. Gel Strength

**Figure 1:**
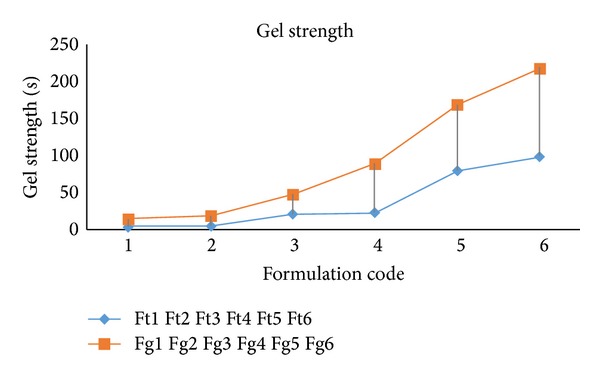
Graphical representation of gel strengths of formulations.

The results obtained for strength test of all the formulations are mentioned in Table 5 and its graphical representation is shown in Figure 1. It has been observed that gel strength increased with the increase in the concentration of mucoadhesive polymer in the formulation. If comparison is made between the Ft and Fg formulations, Fg formulation will show higher gel strength than Ft. The reason can be attributed to the ethanol present in these formulations, as it has a tendency to decrease the gel strength.

### 3.8. Spreadability Test

With increase in the concentration of the polymeric component, viscosity of the solution was increased. At the same time spreadability of the formulation was reduced. This can be observed from the evaluation tests data compiled in Table 5. Ft formulation showed a higher spreadability compared to Fg formulation because gel strength and viscosity of Fg formulation were higher. Consequently, its spreadability was less.

On the basis of gelling capacity, viscosity, gel strength, and spreadability results, formulations Ft1, Ft4, Fg1, and Fg4 showed optimum results within the desired range. So, these six formulations were subjected to further evaluation parameters.

### 3.9. Drug Content

All the formulations reflected fairly uniform drug content ensuring adequacy in the method of preparation of the* in situ* gel. Drug content was found to be within the range of 98.15–99.75%.

### 3.10. *Ex Vivo *Permeation Study

For more details see Table 6.

### 3.11. *In Vitro* Permeation Study

**Figure 2:**
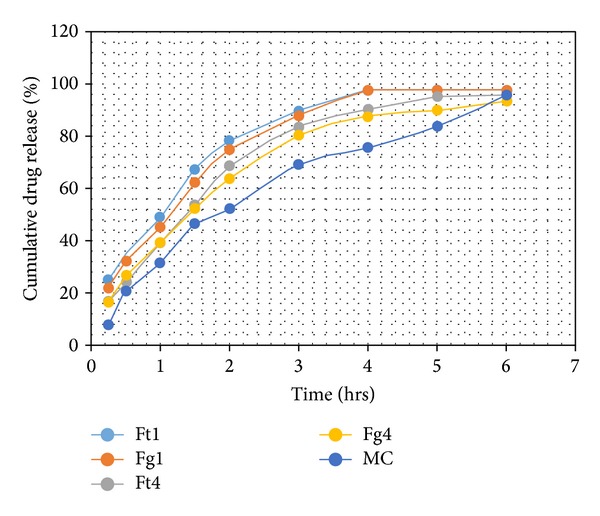
*In vitro* % drug release versus time plot of different formulations.

All the formulations including the marketed cream showed almost similar drug release rates. The drug release rate was high lasting for up to 3 hrs. Thereafter, it remained constant for up to 6 hrs. Initial burst release was higher in* in situ* gel formulations. Among the* in situ* gel formulations, a formulation containing carbopol 934 showed decreased burst release compared to a formulation without carpool 934. Further, kinetic models were applied on these formulations so as to analyze their release mechanism. Kinetic model was also applied in marketed cream (MC) in order to compare the release pattern with that of the prepared formulation (Table 7 and Figure 2).

### 3.12. Mucoadhesion Studies

Mucoadhesion is an important feature of the formulations designed for the delivery of drug in oral cavity. Assessment of the mucoadhesive strength in terms of detachment stress showed that the adhesive properties of gel formulations increased with the increase in the concentration of carbopol 934. The high mucoadhesive strength of the delivery system may lead to prolonged retention and increased absorption across mucosal tissue.

## 4. Conclusion

Temperature and pH sensitive* in situ* gel of ACY (2% w/v) was successfully prepared by cold method using poloxamer 407, carbopol 934, HPMC, tween 80-ethanol (1 : 2 ratio), and glycerin as a drug dissolving solvent.

The gelation temperatures of all the formulations were within the range of 28–38°C. It was observed that the higher the concentration of polymer in the formulation the lower its transition temperature. The formulations containing carbopol 934 showed gelation only when their pH and temperature were raised simultaneously. With regards to the pH, the formulation containing carbopol was slightly acidic then the formulation containing poloxamer. Hence, acidic nature of the carbopol was used to prepare pH triggered* in situ* gel. The pH of carbopol containing formulation was adjusted to pH 5.8, while the pH of poloxamer solution was adjusted to pH 7. By addition or increase in the concentration of hydrophilic polymer, the gelling capacity, gel strength, viscosity, and mucoadhesion were increased whereas spreadability was decreased. All the formulations exhibited fairly uniform drug content. Drug release study of all the formulations showed sustained release properties. The release of drug through these* in situ* gel formulations followed a Higuchi model and Korsmeyer peppas model mechanisms. Delivery of drug through oral mucosa by* in situ* gel formulation avoids the first pass effect. So it is a logical choice for local and systemic delivery of drug, which eventually improves the bioavailability of drug. This approach can be used to treat oral herpes infection locally by improving the patient compliance. However, further studies are needed to be performed, in order to increase the dose of drug in the formulation.

## Figures and Tables

**Table 1:** Composition of the drug loaded formulations containing tween 80 and ethanol.

FC	Drug (gm)	T + E (mL)	P407 (gm)	P188 (gm)	C934 (gm)	HPMC K-100 (gm)	MP (gm)	PP (gm)	D/W (mL)
Ft1	0.6	6	6	—	—	0.15	0.03	0.003	30
Ft2	0.6	6	4	2	—	0.15	0.03	0.003	30
Ft3	0.6	6	4.5	—	0.09	0.15	0.03	0.003	30
Ft4	0.6	6	6	—	0.03	0.15	0.03	0.003	30
Ft5	0.6	6	6	—	0.09	0.15	0.03	0.003	30
Ft6	0.6	6	6	—	0.15	0.15	0.03	0.003	30

Note: FC: formulation code, T: tween 80, E: ethanol, P407: poloxamer407, P188: poloxamer188, C934: carbopol934, HPMC K-100: hydroxypropyl methyl cellulose, MP: methyl paraben, PP: propyl paraben, and D/W: distilled water.

**Table 2:** Composition of the drug loaded formulations containing glycerin.

FC	Drug (gm)	Glycerin (mL)	P407 (gm)	P188 (gm)	C934 (gm)	HPMC K-100 (gm)	MP (gm)	PP (gm)	D/W (mL)
Fg1	0.6	6	6	—	—	0.15	0.03	0.003	30
Fg2	0.6	6	4	2	—	0.15	0.03	0.003	30
Fg3	0.6	6	4.5	—	0.09	0.15	0.03	0.003	30
Fg4	0.6	6	6	—	0.03	0.15	0.03	0.003	30
Fg5	0.6	6	6	—	0.09	0.15	0.03	0.003	30
Fg6	0.6	6	6	—	0.15	0.15	0.03	0.003	30

Note: FC: formulation code, Gly: glycerin, P407: poloxamer 407, P188: poloxamer188, C934: carbopol934, HPMC K-100: hydroxypropyl methyl cellulose, MP: methyl paraben, PP: propyl paraben, and D/W: distilled water.

**Table 3:** Solubility of acyclovir in different pH buffer solutions.

S. no.	Buffer solution	Solubility (microg/mL)
1	HCl buffer of pH1.2	18.315
2	Acetate buffer of pH4.5	10.064
3	Phosphate buffer of pH5.5	2.515
4	Phosphate buffer of pH6.8	2.25
5	Phosphate buffer of pH7.4	2.558
6	Borate buffer of pH9.8	61.842

**Table 4:** Evaluation data 1 of *in situ* gel formulation.

FC	*T*_sol-gel_	Gelling capacity	Appearance		pH	
			Solution state	Gel state	Observed	Adjusted to
Ft1	31.333 ± 1.155	++	*√*	€	8.15	7
Ft2	35.333 ± 1.145	++	*√*	€	8.21	7
Ft3	35.333 ± 0.577	++	*√*	€	5.6	5.8
Ft4	38.666 ± 1.154	++	*√*	€€	5.78	5.8
Ft5	35.666 ± 0.577	++	*√√*	€€	5.34	5.8
Ft6	29.666 ± 1.154	+++	*√√* *√*	€€€	5.56	5.8
Fg1	28.333 ± 0.577	++	∆	○	8.13	7
Fg2	34.666 ± 1.527	++	∆	○	8.18	7
Fg3	37.666 ± 0.577	++	∆	○	5.17	5.8
Fg4	35.333 ± 0.577	++	∆	○○	5.65	5.8
Fg5	31.333 ± 1.155	++	∆∆	○○	5.48	5.8
Fg6	30.333 ± 0.577	+++	∆∆∆	○○	5.63	5.8

+: gel after few minutes dissolves rapidly.

++: immediate gelation remains for few mins.

+++: immediate gelation remains for nearly an hr.

*√*: yellowish solution with less viscosity.

*√√*: yellowish solution with moderate viscosity.

*√√* *√*: yellowish solution with high viscosity.

∆: white colored solution with less viscosity.

∆∆: white colored solution with moderate viscosity.

∆∆∆: white colored solution with high viscosity.

FC: formulation code.

€: yellowish semifluid gel.

€€: yellow colored semistiff gel.

€€€: yellow colored stiff gel.

○: white colored semistiff gel.

○○: white colored stiff gel.

**Table 5:** Evaluation data 2 of *in situ* gel formulation.

FC	Viscosity (Cp)			
	Solution state	Gel state	Spreadability	Gel strength
**Ft1**	**98.93 ± 0.40**	**1481.33 ± 6.80**	**6.63 ± 0.15**	**4.67 ± 0.57**
Ft2	104.88 ± 2.29	1540.67 ± 15.65	6.43 ± 0.05	5.66 ± 0.57
Ft3	149.10 ± 2.73	1505.00 ± 6.00	5.70 ± 0.17	21.33 ± 1.53
**Ft4**	**111.61 ± 10.67**	**1564.00 ± 7.21**	**5.66 ± 0.12**	**23.66 ± 0.57**
Ft5	158.27 ± 6.68	1498.67 ± 14.22	5.26 ± 0.11	80.33 ± 0.57
Ft6	199.74 ± 0.63	1594.33 ± 12.89	4.53 ± 0.12	99.66 ± 0.57
**Fg1**	**214.41 ± 3.08**	**1493.67 ± 5.03**	**6.56 ± 0.05**	**10.33 ± 1.52**
Fg2	220.47 ± 2.16	1495.67 ± 6.65	6.30 ± 0.02	13.66 ± 1.52
Fg3	242.05 ± 2.16	1533.67 ± 19.50	5.66 ± 0.20	26.33 ± 1.52
**Fg4**	**263.60 ± 10.58**	**1586.33 ± 5.50**	**5.03 ± 0.15**	**65.66 ± 1.15**
Fg5	242.50 ± 14.90	1535.33 ± 11.01	5.23 ± 0.05	89.33 ± 0.57
Fg6	344.87 ± 5.56	1621.33 ± 3.21	4.46 ± 0.05	119.3 ± 1.52

The bold data refer to four selected formulations with the best results.

**Table 6:** *Ex vivo* release data of different formulations.

Time (min.)	%Release				
	Ft1	Fg1	Ft4	Fg4	MC
15	25.209	22.203	17	16.584	8.234
30	35.645	32.549	24.625	27.125	20.963
45	49.48	45.489	39.641	39.648	31.839
60	67.457	62.626	53.982	52.637	46.71
75	78.393	75.279	68.892	64.135	52.45
90	89.742	87.993	83.891	80.453	69.45
120	97.638	97.729	90.678	87.825	75.85
180	97.892	97.773	95.365	90.135	83.95
360	97.845	97.715	95.632	93.899	95.87

Fg and Ft: formulation code and MC: marketed cream.

**Table 7:** Kinetic analysis of *in vitro* release data of different formulations.

FC	Zero order		First order		Higuchi model		Hixson crowell model		Korsmeyer peppas model		Best fit model
	*K*	*R* ^2^	*K*	*R* ^2^	*K*	*R* ^2^	*K*	*R* ^2^	*K*	*R* ^2^	
Ft4	16.88	0.91	−0.25	0.904	48.69	0.98	−0.52	0.96	0.58	0.98	Higuchi model
Fg4	15.67	0.91	−0.20	0.911	45.21	0.98	−0.45	0.97	0.56	0.98	Peppas model
Ft1	19.33	0.92	−0.31	0.946	39.94	0.93	−0.56	0.93	0.54	0.97	Peppas model
Fg1	20.22	0.94	−0.31	0.937	42.09	0.94	−0.58	0.93	0.50	0.97	Peppas model
MC	17.66	0.94	−0.20	0.928	43.60	0.99	−0.45	0.98	0.72	0.97	Higuchi model

## Abbreviations

ACY: Acyclovir
HSV: Herpes simplex virus.
